# Telenursing: Bioinformation Cornerstone in Healthcare for the 21st Century

**DOI:** 10.6026/97320630013412

**Published:** 2017-12-31

**Authors:** Nicole Balenton, Francesco Chiappelli

**Affiliations:** 1 Oral Biology & Medicine, School of Dentistry, Center for the Health Sciences University of California Los Angeles, USA;; 2 Health Sciences, California State University, Northridge, USA;; 3 Evidence-Based Decisions Practice-Based Research Network, USA;

**Keywords:** Bioinformation, tele-health, tele-nursing, evidence-based practice, drone emergency kits

## Abstract

Bioinformation is at the very core of 21st-century healthcare. Telehealth consists of the range of healthcare-related services delivered through bioinformation-aided telecommunications across health-related disciplines, including nursing. Whereas it is clear that bedside patient-centered nursing can never be replaced, recent developments in bioinformation-aided telenursing will undoubtedly contribute to improving healthcare effectiveness and efficacy. Current trends show that as telenursing becomes increasingly timely and critical, healthcare professionals adopt new and improved evidence-based practices as a standard for patient care worldwide.

## Background

Bedside nursing will never be replaced. Nonetheless, nursing is being enhanced with today's cutting-edge technology and the continuous generation of bioinformational developments and advancements. By contrast, the medical field often lags behind when implementing these new and improved healthcare practices. Healthcare strives to provide the optimal effectivenessfocused, patient-centered, and evidence-based practices for patients to ensure the highest quality of care [1, 2], the ongoing debate of merging medicine and technology remains ongoing in the United States (US) and worldwide. The US alone spent close to $3.3 trillion on healthcare in 2016 [3], and continues to struggle to achieve healthcare goals or address issues of accessibility, quality, and cost for all citizens.

The growing geriatric population and the rising number of the uninsured could aggravate this pattern, unless concerted actions by the healthcare system are directed to bring about necessary improvements. Compounding this problem is a rapidly changing growing nursing shortage in the US predicted for the next decade. Healthy People 2020, a 10-year national initiative focused on improving the health of all Americans, works toward the goal of improving health care services overall, despite the gargantuan obstacles [4].

Current trends in telehealth indicate that telenursing can significantly attenuate these difficulties by redesigning healthcare practices and improving the delivery of quality care [4]. Telehealth includes a range of bioinformation-based services delivered through telecommunications across all health-related disciplines ranging from pharmacology, radiology, psychology, medicine, and nursing [5]. The Health Resources and Services Administration (HRSA) of the US Department of Health and Human Services defines telehealth as the ensemble of telecommunication technologies that "support and promote longdistance clinical health care, patient, and professional health-related education, public health, and health administration" [6, 7]. Technologies include the Internet, media, and video or webbased conferencing.

The need for remote care began in early periods where early forms of technology and communication interconnected medical sites for treating the sick and elderly. The momentum of telehealth significantly advanced when the demand for telemedicine took notice among the American National Aeronautics and Space Administration (NASA) in the 1990's [8]. NASA and medical professionals in the US jointly created telemedicine as a novel means to monitor the health of astronauts in space [8]. The technology continued to flourish as the development of the Internet progressed, and information and communication technologies (ICT) established itself as bioinformation, a new disciple in its own right [6, 9].

Telenursing emerged as an important branch of telemedicine: the practice of telehealth and technology used together to optimize nursing care for patients and populations in remote locations [10, 11]. Successful telenursing involves complex computer-based systems that utilize video and audio features integrated with medical monitoring systems. Through these modes, nurses provide immediate, ongoing care, and can better consult their patients leading to improve clinical and health service outcomes. Together with information technology, telecommunication and bioinformation in general, nurses now bring quality care regardless of geographical location or distance, maximizing effectiveness, efficacy and efficiency.

Developments in telenursing incorporate innovations in bioinformational technology. New devices assist telenursing practice, and are continually enhanced to improve the delivery of nursing care through telenursing: case in point, telenursing kits, which bring healthcare services into the comfort of the patient's home, target the chronically ill underserved populations, geriatric patients and other special patient populations by supplying devices that can collect biometric data, interpret results and monitor patients to ensure that they comply with their discharge orders and treatment plan. It is self-evident that these kits are most effective when they are used in conjunction with auxiliary bioinformation tools, such as telenursing computers equipped with audio and visual features, and further optimized when nurses can perform physical exams, run the necessary diagnostic tests and assessments, obtain informed consent, and provide all the informational materials patients and stakeholders might need in real-time. These kits provide the ability to perform these nursing activities anytime and anywhere the patient is, from the isolated Asian rice fields to the Amazonian pauper villages, to the inner cities of our metropolis.

**Figure 1:**
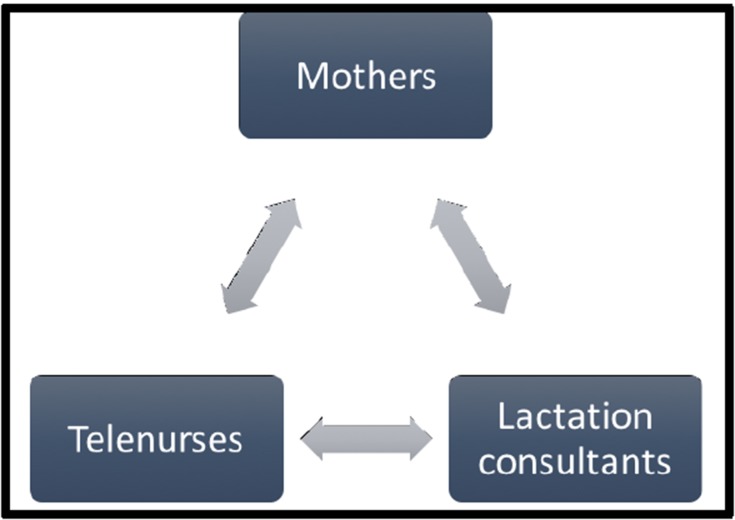
Model of Telenursing for Peri-Natal Care and Breastfeeding

Telenursing kits used in the comfort of the patient's home also provide a more convenient protocol, which ensure privacy for the handicapped and the geriatric patient population using private methods. Among rural and underserved populations, telenursing and telecommunications encourage healthy behavior practices, including peri-natal care and breastfeeding (Figure 1).

Through telecommunication, nurses and lactation consultants educate new mothers, family members and stakeholders about the benefits of breastfeeding, and guide them on proper lactation techniques. New mothers receive audio-visual materials and other guided technology, which guide them to replicate the best breastfeeding positions for both mother and baby. Through telenursing, mothers and stakeholders increase their health literacy with respect to the necessary knowledge skills, and selfefficacy to ensure optimal breastfeeding practices.

Another important new modality of telenursing involves drone emergency responses. The emergency medical system release drones to the target location with telehealth and bioinformation modalities, which can anticipate the arrival of specialized trauma teams. During an emergency health crisis, nursing is critically needed but often impossible to dispense in hard-to-reach locations. Drones are the most efficient way nurses can provide their care in those situations. Drones travel distances faster and reach more safely locations that emergency medical personnel often cannot access.

Drones are equipped with easy-to-use medical technology devices such that any adult can safely perform basic, life-saving procedures, can efficiently deliver emergency kits with critical supplies. Drone emergency kits include medical supplies such as first aid, automated external defibrillator, and high-tech camera glasses to enable the victims to communicate with emergency care nurses, who can evaluate the medical emergency through the specialized lenses and provide appropriate directions for care.

A third current and critical bioinformational modality of telenursing consists of portable mobile healthcare. Applications include video-audio conference calls, messenger chats, as well as in-app appointment bookings. Smartphone applications through telenursing allow patients, especially those with chronic illnesses and mental health conditions, to obtain ongoing nursing assessment and monitoring. Patients have immediate accessibility to track their health reports, treatment plan, and progress while being away from the hospital or home, and, as importantly, to communicate with the nurse. Medical smartphone applications ensure patient privacy as well as advanced comfortable care.

## Conclusion

In conclusion, whereas it is incontrovertible that technological advancement will never replace the work of nurses play at the bedside, trends establish that telenursing is an evolving bioinformation-based tool that improves the nursing practice by bringing the nurses' skills and knowledge to patients who are out of physical reach. As pivotal figures in the progression and continued development of telenursing, nurses are at the forefront of delivering optimal effectiveness-focused, patient-centered, and evidence-based clinical services. Telenursing has significant potential to establish, integrate, validate and standardize new and improved bioinformation-based healthcare modality in the next decade [9, 11].

Expectations are that telenursing will benefit the future of medicine in general, and of nursing in particular with respect to the delivery of quality patient care. Telenursing will expand its reach among nurses in hospitals, triage centers, rehabilitation facilities, home-health agencies, and disease-management companies [10, 11]. Bioinformational technological methods, including interactive voice and video calls will increasingly allow nurses and patients to see, communicate and interact with each other. This value-added facet of telenursing will permit nurses to extend their practice beyond medical care settings, and offer patients aid where healthcare may not be as advanced or accessible.

Telenursing will redesign the nursing field by making it more effective and safe. Its advantages will echoe across the healthcare system allowing nursing professionals to adopt evidence-based practices as a standard for patient care. Evidence-based practice will significantly impact the future of nursing practice, education, and science for years to come [10, 11]. It is possible and even probable that telenursing will offer a positive change in nursing and an opportunity for healthcare to flourish and grow alongside technological advancements. Incoming nurses and health professionals to the field will have that level of support from telenursing and evidence-based practices that will help make their transition to the field much smoother.

In brief, current data support many of the benefits of tele-nursing, including increasing access to care, patient adherence, monitoring patient safety, technological advancements, and allowing healthcare providers to network with one another [5, 6, 9, 10]. Advancements of telenursing are expected to confirm and expand these current trends in the next decades, and contribute to the establishment of telenursing as a vital and cutting-edge nursing practice in the US and worldwide to ensure patientcentered evidence-based healthcare.
